# Effect of a Reminder System on Pre-exposure Prophylaxis Adherence in Men Who Have Sex With Men: Prospective Cohort Study Based on WeChat Intervention

**DOI:** 10.2196/37936

**Published:** 2022-08-11

**Authors:** Bing Lin, Jiaxiu Liu, Wei He, Haiying Pan, Yingjie Ma, Xiaoni Zhong

**Affiliations:** 1 School of Public Health and Management Chongqing Medical University Chongqing China; 2 Research Center for Medicine and Social Development Chongqing China; 3 School of Medical Informatics Chongqing Medical University Chongqing China

**Keywords:** pre-exposure prophylaxis (PrEP), adherence, reminder system, men who have sex with men (MSM), WeChat, oral PrEP, HIV prevention, MSM, reminder, message

## Abstract

**Background:**

The efficacy of pre-exposure prophylaxis (PrEP) is highly dependent on adherence, and one of the main reasons for poor adherence is forgetfulness. Therefore, it is important to explore how to remind users to take their medicine on time.

**Objective:**

This study aims to explore the effect of a reminder system on PrEP adherence in men who have sex with men (MSM) to improve adherence. The main function of the reminder system based on the WeChat social media app is to send daily messages to PrEP users reminding them to take their medicine.

**Methods:**

An open-label, multicenter, prospective cohort study of PrEP in HIV-negative MSM was conducted from November 2019 to June 2021. Study participants who met the criteria were randomly divided into 2 groups: no-reminder group and reminder group. Both groups received daily oral PrEP with follow-up every 3 months. Adherence was measured on the basis of self-report and was defined as the percentage of medications taken on time. Participants in the reminder group scanned a WeChat QR code and received a reminder message every day. Participants in the no-reminder group took daily oral medicines without reminders. The longitudinal trajectories of adherence for both groups were displayed to compare the variability in adherence at each time point. The association between the changes in adherence (no change, improvement, decline) at each time point and the use of the reminder system was analyzed by multinomial logistic regression models to further explore the effectiveness of the system.

**Results:**

A total of 716 MSM were included in the analysis, that is, 372 MSM in the no-reminder group and 344 MSM in the reminder group. Adherence in the no-reminder group fluctuated between 0.75 and 0.80 and that in the reminder group gradually increased over time from 0.76 to 0.88. Adherence at each time point was not statistically different between the 2 groups. Further analysis showed that an improvement in adherence in the early stage was associated with the use of the reminder system (odds ratio [OR] 1.65, 95% CI 1.01-2.70; *P*=.04). An improvement in average adherence compared to initial adherence was positively associated with the use of the reminder system (OR 1.82, 95% CI 1.10-3.01; *P*=.02).

**Conclusions:**

The effect of the reminder system on PrEP adherence in MSM was more significant in the early stage, which is related to the increased motivation of users and the development of medicine-taking habits. The reminder system is potentially effective for early-stage medicine management, encouraging users to develop healthy medicine-taking habits and to increase their adherence.

**Trial Registration:**

Chinese Clinical Trial ChiCTR190026414; http://www.chictr.org.cn/showproj.aspx?proj=35077

## Introduction

Globally, men who have sex with men (MSM) bear a disproportionate burden of HIV, and they are a high-risk group for infection [1]. In China, more than a quarter of new HIV diagnoses can be attributed to MSM [2]. Despite various interventions, the risk of HIV infections among MSM in China is increasing, with the proportion of new HIV infections rising from 2.5% to 25.5% [3,4]. Given the existing circumstances, several studies have been exploring ways to prevent HIV infection in MSM populations, including biomedical interventions such as oral pre-exposure prophylaxis (PrEP) [5,6]. PrEP is a biological HIV prevention intervention that focuses on reducing the risk of HIV infection through daily (or event-based) oral antiretroviral medication [7]. In fact, several clinical trials and cohort studies have demonstrated the safety and efficacy of oral PrEP in reducing the risk of HIV infection in MSM [8]. However, the efficacy of PrEP for HIV prevention is highly dependent on adherence [9,10].

PrEP advocates and researchers agree that one of the important issues that needs to be addressed before PrEP can be scaled up is the concern about nonadherence [11,12]. As a result, researchers have focused their attention on PrEP adherence and the factors that influence it. Prior studies have shown that the potential barriers to daily oral PrEP include living arrangement, side effects, stigma, and forgetfulness [13,14]. In addition, another study found that forgetting to take medicines is the most common objective reason for nonadherence, accounting for 70.21% [15]. Taken together, forgetfulness is one of the main reasons for poor adherence, and reminding users to take medicines is an important way to improve adherence. Exploring how to obtain and maintain high adherence in MSM populations is crucial for promoting the effective implementation of PrEP intervention strategies and reducing new HIV infections.

Different methods can be considered to remind MSM to take their medicines on time. Many attempts have been made in this direction by researchers. Mobile health (mHealth) technologies are effective and cost-effective strategies to improve individual and public health [16,17]. Among them, SMS text messaging is often used to help remind users to take their medicines, which can improve adherence, owing to its ubiquity and ease of use in mobile devices. The feasibility and acceptability of SMS text messaging as a potential tool for primary HIV prevention has been demonstrated [18,19]. Meanwhile, other studies have also developed a novel mobile app to support PrEP adherence through artificial intelligence and an electronic sexual diary, which have received positive feedback from users, thereby providing a further basis for future effectiveness studies [20]. These studies of improving adherence through a “reminder function” have been tested in Africa, Thailand, Peru, and the United States, but implementation in China is uncommon [21,22].

Like Facebook and Twitter, WeChat is a popular social media app in China. According to the data analysis, WeChat is the most popular social media platform with over 1 billion registered users [23]. Approximately 93% of the residents in the major Chinese cities log on to WeChat every day [24]. The high ownership of mobile phones and the widespread popularity of the WeChat app suggest that this is a promising platform for providing low-cost interventions. Moreover, WeChat-based interventions have shown feasibility and acceptability in HIV prevention and control in China [25,26], which includes MSM [23]. However, studies using the WeChat app to remind MSM to take their medicine daily during PrEP use are limited in China.

Therefore, we conducted a prospective cohort study of PrEP adherence in the MSM population in Western China. Our reminder system is based on the WeChat app for mobile phones that scans individual QR codes and connects to the backend management system to send daily reminder messages. We compared the variability of adherence between the no-reminder group and the reminder group at each time point by plotting the longitudinal trajectory of adherence. At the same time, the relationship between changes in adherence (no change, improvement, decline) and the use of the reminder system was investigated to confirm the influence of the reminder system on adherence and to provide a theoretical foundation for the improvement of the reminder system in the future so as to improve adherence and increase the effectiveness of PrEP.

## Methods

### Ethics Approval

All procedures of this study were in accordance with the ethics approval granted by the ethics committee of Chongqing Medical University (2019001). The ethics committee of Chongqing Medical University has reviewed the proposed use of human participants in the abovementioned project.

### Data Collection

This study was a PrEP open-label, multicenter, prospective cohort study conducted from November 2019 to June 2021 in 3 regions of Western China: Chongqing, Sichuan, and Xinjiang (Chinese clinical trial registration ChiCTR190026414). MSM who met the criteria were recruited through collaboration with local nongovernment organizations and peer recommendations. Inclusion criteria for the study population were (1) physiological male (assigned male sex at birth), (2) older than 18 years, (3) had engaged in sex with male partners in the past 6 months, (4) negative HIV antigen-antibody test, (5) willing to use medicines under guidance and subject to follow-up arrangements, and (6) signed informed consent form.

**Figure 1:**
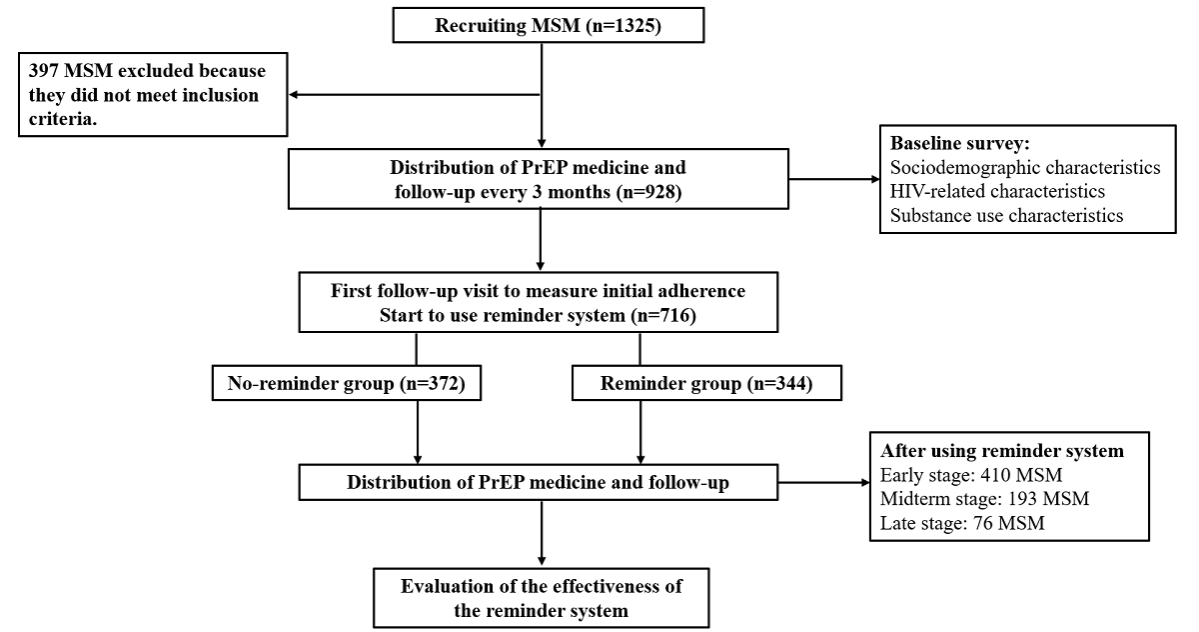
Flowchart of the recruitment, survey, and follow-up of the study participants. MSM: men who have sex with men. PrEP: pre-exposure prophylaxis.

The MSM population was screened for inclusion criteria and then divided into 2 groups: no-reminder group and reminder group. After completing the baseline survey, participants were followed up every 3 months and given their medicines by our study researchers. They received daily oral dose of Lamivudine and Tenofovir Disoproxil Fumarate tablets (300 mg/tablet). The first 3 months after joining the cohort were considered as an observation period with no use of the reminder system. The initial adherence of the study participants was measured and the reminder system was started at the first follow-up visit. Participants in the reminder group scanned a WeChat QR code (unique identity QR code, scanned only once) to receive daily medicine reminder messages, while the no-reminder group took daily oral medicines without reminders. We designated the intervals corresponding to each follow-up time point as early, midterm, and late stages after the initial follow-up visit (beginning to employ the reminder system). A total of 716 MSM were included in the analysis: 372 MSM in the no-reminder group and 344 MSM in the reminder group. Figure 1 presents the flow chart of the recruitment, survey, and follow-up of the study participants.

### Reminder System of PrEP

**Figure 2:**
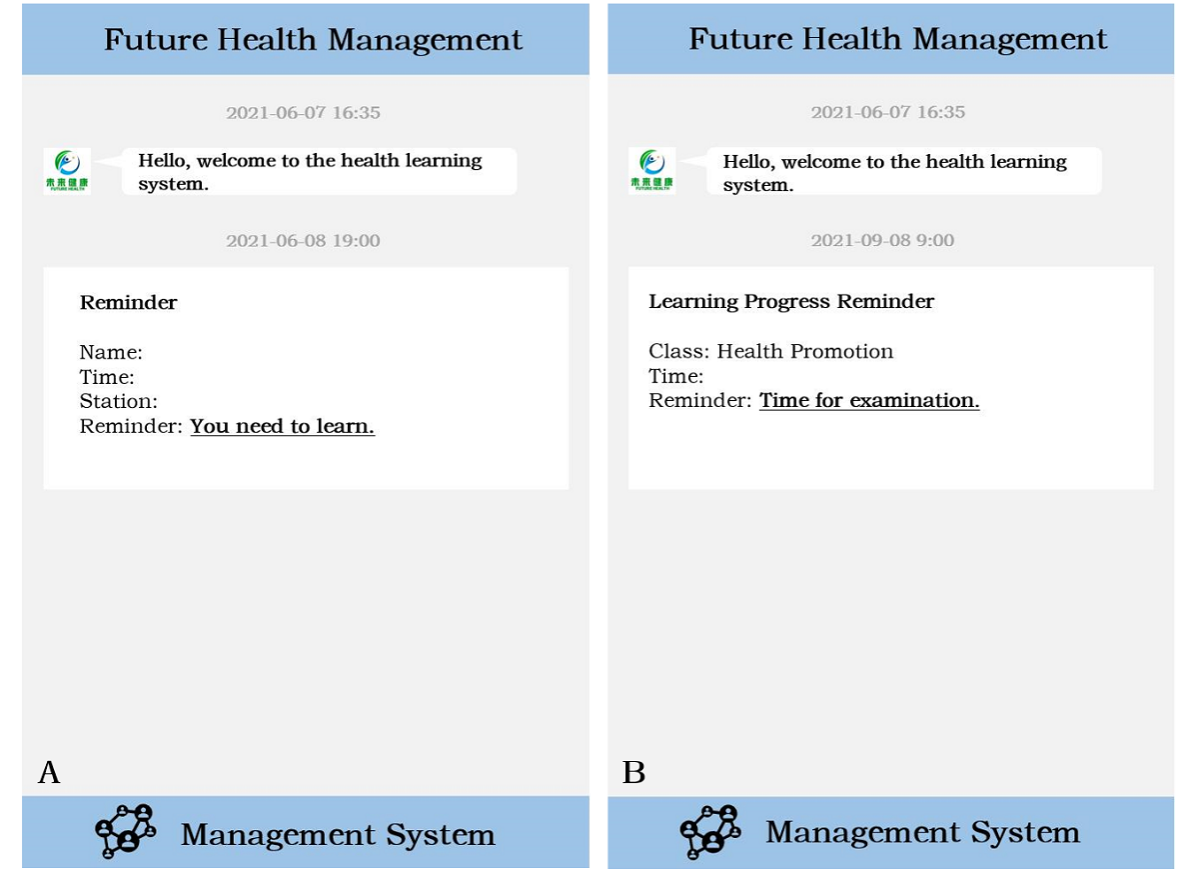
A: reminds users that they should take their medicine that day and B: reminds users that they should go for a follow-up visit.

Our study developed a PrEP reminder system in conjunction with Chongqing Future Health Management Company Limited. This system relies on the internet and intelligent medicine information tracking management system using cloud computing, big data, intelligent hardware, and other new generation information technology products to provide effective medicine management for the study participants through the process of medicine-taking plan, medicine reminder, and health knowledge promotion to achieve the expected prevention effect. Our researchers typed in the basic information of the reminder group into the background management system and generated an independent personal QR code. Participants were bound to the backend system after scanning the QR code using their WeChat apps. Daily reminders were sent from the next day, and users could set the time according to their request. In order to protect the privacy of the users, the content of our messages was relatively obscure. For example, when users received a “You need to learn” message from Future Health Management, it is a reminder that they should take the medicine that day. When users received a “Time for examination” message from Future Health Management, it is a reminder that they should visit the study center for the 3-month follow-up (Figure 2).

### Baseline Variables

The baseline survey included demographic characteristics, HIV-related characteristics, and substance use characteristics. Among them, the demographic characteristics include age, household register location, education attainment, employment status, marital status, and monthly personal income. HIV-related characteristics mainly included HIV testing and counseling, number of sexual partners, condom use, and HIV risk perception. Participants were asked, “In the last month, how many male sexual partners (casual and regular) have you had? How many times did you have sex with a male sexual partner? How many of these times did you use a condom?” These questions were used to measure the number of male sexual partners and condom use during sex. We defined that condoms were used at each sexual intercourse and if the number of condom use was greater than or equal to the number of sexual intercourses. At the same time, participants were asked, “How likely do you think you are to get AIDS?” We used this question to measure the participants’ perceived HIV risk. Participants responded on a scale of 1-5, with a score of 1 indicating very small and 5 indicating very large. We defined a score ≥3 as the subgroup with higher perceived HIV risk. Substance use mainly included alcohol use. Participants were asked, “in the last month, how often did you drink alcohol?” Participants responded, “basically every day,” “at least 3 times a week,” “at least 1 time a week,” “less than 1 time a week,” or “never drink alcohol.” We divided the alcohol use into 2 categories: one for never drinking and one for ever drinking.

### Adherence

**Figure 3:**
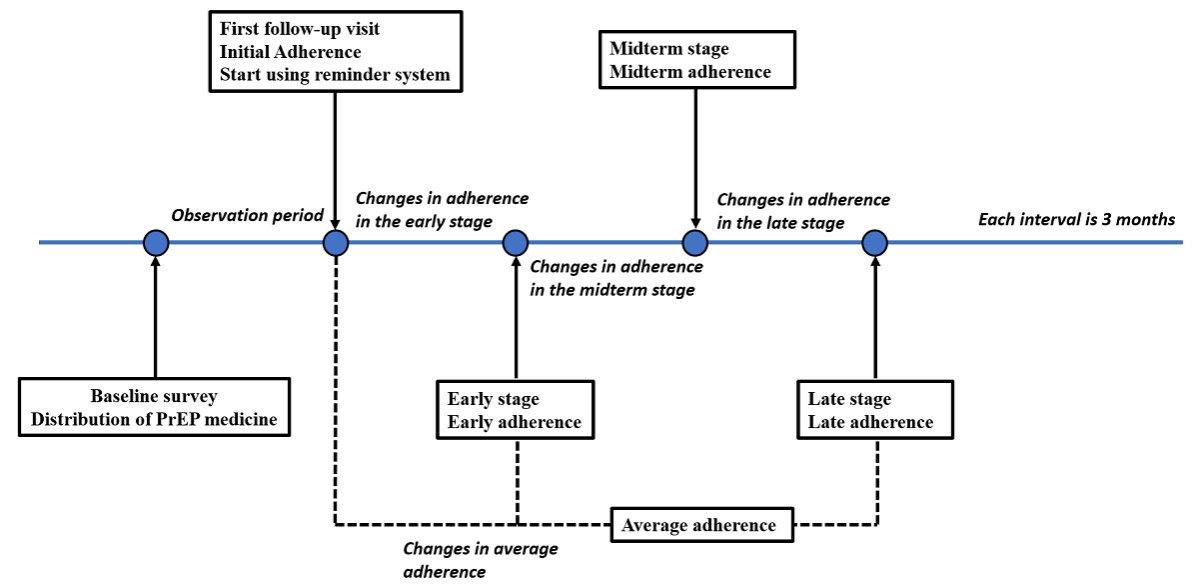
Explanation of adherence (initial adherence, early stage adherence, midterm adherence, late stage adherence, average adherence) and changes in adherence by period. PrEP: pre-exposure prophylaxis.

At each follow-up visit, adherence was measured by self-report. Study participants were asked, “in the last two weeks, have you missed any doses? how many days did you miss?” Participants answered “yes” or “no” and the number of days missed (0-14 days). Adherence was equal to the percentage of days adhered. All answers were checked by our researchers. If illogical answers occurred, quality control and corrections were made in the study site. After completing the baseline survey, participants entered an observation period, taking their medicines without reminders. The participants arrived for the first follow-up visit 3 months later, at which point adherence was referred to as “initial adherence.” In the meantime, study participants in the reminder group began using the reminder system. After this, adherence during the follow-up period was defined as early stage adherence, midterm adherence, and late stage adherence. Changes in adherence at each follow-up time point (no change, improvement, decline) were compared to the adherence in the previous period. For example, the changes in adherence in the early stage is defined as early stage adherence minus initial adherence. Average adherence is the mean of adherence in the early, midterm, and late periods, and changes in average adherence is defined as average adherence minus initial adherence. The definition of adherence in each period is shown in Figure 3.

### Statistical Analyses

We compared the variability of the baseline demographic characteristics, HIV-related characteristics, and substance use characteristics between the no-reminder group and reminder group of the MSM population. Trajectories of adherence were plotted for the 2 groups. The nonparametric test (Kruskal-Wallis test) was used to compare the variability of adherence between the 2 groups at each follow-up time point. A multinomial logistic regression model was developed using the changes in adherence (no change, improvement, decline) in each period of follow-up as the dependent variable, and the relationship between the reminder system and the changes in adherence was further assessed by odds ratio (OR) and 95% CI. Variables with *P*≤.15 were first screened by one-way analysis of variance, which was included in the multinomial logistic regression model. The efficacy of the reminder system was assessed by exploring the factors influencing the improvement and decline of adherence, using no change in adherence as the reference. *P*<.05 indicated a statistical difference. All statistical analyses were performed with SAS software (SAS Institute).

## Results

A total of 716 MSM were included in the analysis, with 372 in the no-reminder group and 344 in the reminder group. The baseline demographic characteristics, HIV-related characteristics, and substance use characteristics of the 2 groups were not statistically different in one-way analysis of variance. The results of the descriptive analyses and chi-square tests for each variable are shown in Table 1.

**Figure 4:**
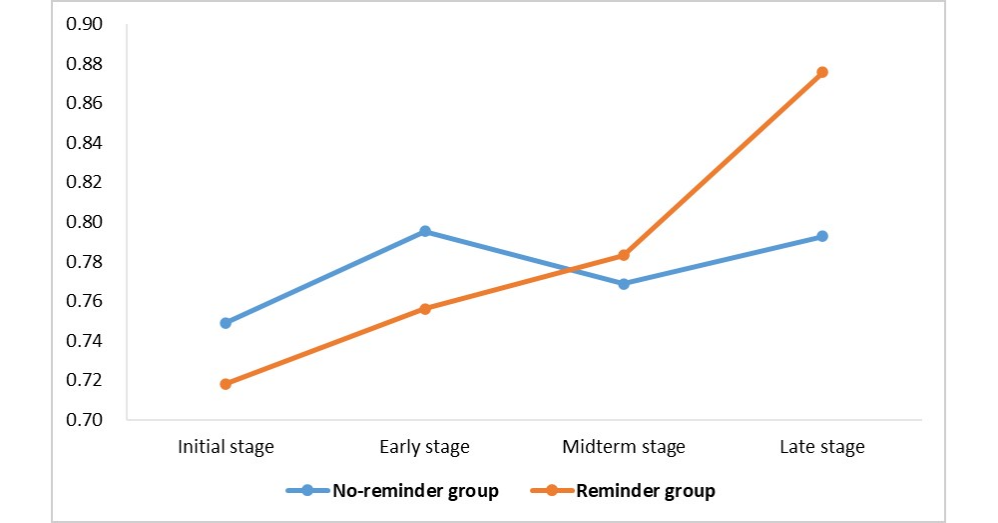
Trajectory plot of adherence at each time point in the men who have sex with men population in the no-reminder group and reminder group.

Adherence trajectories were plotted for the no-reminder group and reminder group (Figure 4). The median of initial adherence was 1.00 (IQR 0.64-1.00) in the no-reminder group and 1.00 (IQR 0.47-1.00) in the reminder group, with no statistical difference in initial adherence between the 2 groups (*P*=.48). After using the reminder system, adherence in the no-reminder group fluctuated between 0.75 and 0.80; adherence in the reminder group gradually increased over time from 0.76 to 0.88. There was no statistical difference in adherence between the 2 groups at the early (*P*=.69), midterm (*P*=.96), and late (*P*=.37) stages of follow-up. After using the reminder system, the mean adherence for the 3 periods in the no-reminder group was 0.79 and the mean adherence in the reminder group was 0.78, which was not statistically different (*P*=.82) (Table 2).

**Table 1:** Differences in the baseline demographic characteristics, HIV-related characteristics, and substance use characteristics between the no-reminder group and reminder group in the men who have sex with men population (N=716).

Variables		No-reminder group (n=372), n (%)	Reminder group (n=344), n (%)	*P* value	
**Age (years)**					.93
	18-30	165 (44.47)	149 (43.31)		
	31-45	158 (42.59)	151 (43.90)		
	>45	48 (12.94)	44 (12.79)		
**Household register location^a^**					.52
	Urban	254 (69.21)	245 (71.43)		
	Rural	113(30.79)	98 (28.57)		
**Education attainment^a^**					.87
	Junior high school and below	27 (7.28)	27 (7.85)		
	High school	84 (22.64)	70 (20.35)		
	College	94 (25.34)	93 (27.03)		
	Undergraduate training or higher	166 (44.74)	154 (44.77)		
**Employment status^a^**					.54
	Employed	307 (82.75)	275 (79.94)		
	Internal student	34 (9.16)	40 (11.63)		
	Jobless	30 (8.09)	29 (8.43)		
**Marital status^a^**					.45
	Married	50 (13.51)	53 (15.50)		
	Single	320 (86.49)	289 (84.50)		
**Monthly personal income^a^ (¥1=US $0.15)**					.41
	≤¥1000	25 (6.79)	22 (6.40)		
	¥1000-¥3000	59 (16.03)	71 (20.64)		
	¥3000-¥5000	125 (33.97)	104 (30.23)		
	>¥5000	159 (43.21)	147 (42.73)		
**HIV testing^a^**					.78
	Yes	341 (92.41)	317 (92.96)		
	No	28 (7.59)	24 (7.04)		
**HIV counseling^a^**					.37
	Yes	267 (71.97)	257 (74.93)		
	No	104 (28.03)	86 (25.07)		
**Number of male sexual partners in the last month^a^**					.50
	0	38 (10.33)	27 (7.99)		
	1	204 (55.43)	198 (58.58)		
	≥2	126 (34.24)	113 (33.43)		
**Condoms were used at each sexual intercourse^a^**					.65
	Yes	301 (83.84)	279 (82.54)		
	No	58 (16.16)	59 (17.46)		
**Finding sex partners through the internet**					.67
	Yes	250 (67.20)	226 (65.70)		
	No	122 (32.80)	118 (34.30)		
**HIV risk perception^a^**					.79
	High	156 (42.05)	148 (43.02)		
	Low	215 (57.95)	196 (56.98)		
**If your male sexual partners know you are using pre-exposure prophylaxis, their attitude is**					.08
	Positive	174 (46.77)	156 (45.35)		
	Neutral	151 (40.59)	160 (46.51)		
	Negative	47 (12.63)	28 (8.14)		
**Have you ever been diagnosed with a sexually transmitted disease by a doctor^a^**					.18
	Yes	18 (4.89)	25 (3.73)		
	No	350 (95.11)	317 (92.69)		
**Commercial sex^a^**					.83
	Yes	15 (4.05)	15 (4.39)		
	No	355 (95.95)	327 (95.61)		
**Alcohol use in the last month**					.45
	Yes	217 (58.33)	191 (55.52)		
	No	155 (41.67)	153 (44.48)		

^a^Indicates missing data.

**Table 2:** Differences in adherence at each time point among the men who have sex with men population in the no-reminder group and reminder group.

		No-reminder group				Reminder group				*P* value^a^
		n	Mean (SD)	Median (IQR)	n		Mean (SD)	Median (IQR)		
Initial adherence (N=716)		372	0.75 (0.37)	1.00 (0.64-1.00)	344		0.72 (0.39)	1.00 (0.47-1.00)	.48	
**After using the reminder system**										
	Early stage adherence (n=410)	217	0.80 (0.33)	1.00 (0.79-1.00)	193		0.76 (0.37)	1.00 (0.64-1.00)	.69	
	Midterm adherence (n=193)	100	0.77 (0.37)	100 (0.68-1.00)	93		0.78 (0.35)	1.00 (0.79-1.00)	.96	
	Late stage adherence (n=76)	41	0.79 (0.31)	1.00 (0.64-1.00)	35		0.88 (0.23)	1.00 (0.79-1.00)	.37	
	Average adherence (n=410)	217	0.79 (0.31)	0.93 (0.72-1.00)	193		0.78 (0.32)	0.97 (0.64-1.00)	.82	

^a^Kruskal-Wallis test was used.

**Figure 5:**
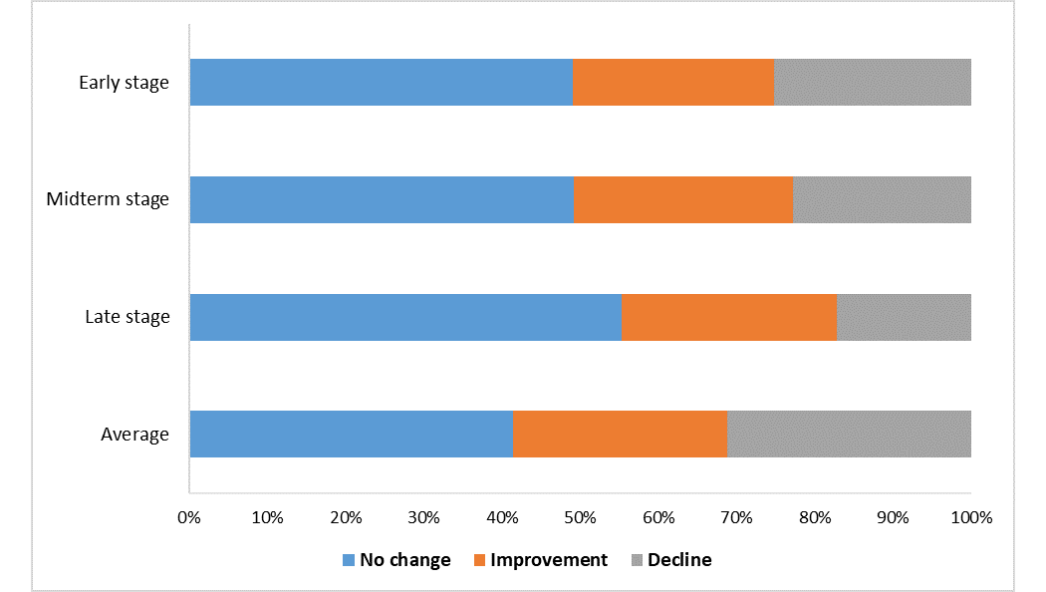
Distribution of changes in adherence.

To further explore the effect of the reminder system on adherence, the percentage of changes in adherence (no change, improvement, decline) at each time point in the MSM population is shown in Figure 5. A percentage bar graph was used to show the distribution of the changes in adherence. No change in adherence was used as a reference for the dependent variable, and the no-reminder group was used as a reference for the independent variable. After the one-way analysis of variance, variables with *P*≤.15 were included in a multinomial logistic regression model for adjusting. The adjusted variables in the early stage included age, HIV counseling, and male sexual partners’ attitude. The adjusted variables in the midterm stage included age, household register location, education attainment, male sex partners, and their attitudes. The adjusted variables in the late stage included household register location and male sexual partners’ attitude. In the model for changes in the average adherence, the adjusted variables included age, HIV testing, HIV risk perception, alcohol use, and male sexual partners’ attitude. After adjusting for basic demographic characteristics, HIV-related characteristics, and substance use characteristics with no change in adherence as a reference, analysis based on multinomial logistic regression models was obtained (Table 3). An improvement in adherence in the early stage was positively associated with the use of the reminder system (OR 1.65, 95% CI 1.01-2.70; *P*=.04). The result showed that the reminder system was not able to prevent a decline in adherence in the early stage, and the difference was not statistically significant (*P*=.78). The changes in adherence in both the midterm and late stages were not statistically associated with the reminder system (*P*>.05). An improvement in the average adherence compared to the initial adherence was associated with the use of the reminder system (OR 1.82, 95% CI 1.10-3.01; *P*=.02); a decline in the average adherence was not associated with the reminder system (*P*=.67).

**Table 3:** Multinomial logistic regression analysis of the changes in adherence at each time point after using the reminder system.

Time point, variables			Improvement versus no change				Decline versus no change		
			Odds ratio (95% CI)		*P* value		Odds ratio (95% CI)		*P* value
**Early stage**									
	Reminder group	1.65 (1.01-2.70)		.04		0.93 (0.57-1.53)		.78	
	No-reminder group	Reference		Reference		Reference		Reference	
**Midterm stage**									
	Reminder group	1.43 (0.67-3.03)		.36		1.01 (0.46-2.24)		.98	
	No-reminder group	Reference		Reference		Reference		Reference	
**Late stage**									
	Reminder group	0.73 (0.23-2.26)		.58		0.16 (0.03-1.03)		.054	
	No-reminder group	Reference		Reference		Reference		Reference	
**Average**									
	Reminder group	1.82 (1.10-3.01)		.02		0.90 (0.55-1.48)		.67	
	No-reminder group	Reference		Reference		Reference		Reference	

## Discussion

### Principal Findings

Our study explored the effect of a reminder system on PrEP adherence to promote adherence and support HIV prevention efforts in the MSM population. We found that the reminder system contributed to an improvement in adherence in the early stage. Previous studies have mentioned that text message reminders are one of the interventions that can improve adherence [27]. Two pilot studies and a PrEP demonstration project in Kenya and Brazil have also demonstrated that incorporating text message reminders into PrEP practices is an acceptable method [28-30]. These reminder methods have been found to be effective in improving adherence by increasing the user’s knowledge of the medicine, motivation, and perceived support through text messages [31]. Meanwhile, according to Rogers’ Protective Motivation Theory, users’ motivation factors may be the driving force for individual behavior change [32]. A prior study has also confirmed that the Protective Motivation Theory can provide a valuable reference for the study of PrEP adherence in the MSM population [33]. The newly developed reminder system in our study was based on text message, and the presentation of this information can increase users’ motivation to take their medicines and encourage them to change their behavior, especially in relation to PrEP adherence.

We believe that the use of the reminder system can encourage users to form medicine-taking habits. Habit is defined as behavior that is performed subconsciously without thought [34]. In the book of “The Power of Habit,” Charles Duhigg defines habit as a 3-step loop of cue, routine, and reward [35]. Habits are behaviors that are induced by situational cues, and our reminder system serves as a cue. Previous findings have shown that habit formation interventions have the potential to improve adherence to antiretroviral therapy [36]. In the meanwhile, as mentioned in the previously published review, the idea of “habit” has been applied to the Medication Usage Skills for Effectiveness program [35]. In their program, users selected cues to help them remember to take their medicine, such as a specific time, a meal, or a reliable daily ritual. According to the findings of that study, the reminder system provided daily cues to users, motivating them to develop the habit of taking medicine and improving their adherence. However, the reminder system was ineffective in preventing adherence declines in the early stage as well as changes in adherence in the midterm and late stages of follow-up. We believe that there are several main reasons for this.

First, according to previous survey results, 70.21% of MSM did not take their medicines because they forgot, while 29.08% said they were too busy, 28.01% were worried about the side effects, and 18.44% thought it was troublesome to take medicine [15]. Forgetting to take medicine is the main factor affecting adherence, but there are still other factors that can potentially impact adherence. Reminder systems are primarily targeted at MSM who forget to take their medicine, and the effect of reminder systems on adherence may not be significant for those users with low willingness to take medicine. Therefore, reminder systems are ineffective in stopping the decline in adherence in MSM in the early stage.

Second, as we mentioned before, practice over many repetitions can help develop habits. The increased level of early stage adherence may be related to the reminder system that motivates users to develop the habit of taking medicine. However, repetitive activity eventually transforms the individual’s cognitive control from a conscious to an automatic process [37]. This suggests that once a habit is formed, the act of taking medicines shifts from a conscious motivated behavior to an unconscious automatic one, which does not require external reminders. This may lead to a less effective reminder system in the midterm and late stages. Therefore, if the reminder system is used for medicine management in the early stage, it can not only promote the formation of medicine habits and improve adherence among users but also make the use of reminder systems more targeted and effective.

In addition, while most participants expressed positive attitude toward using the reminder system, a few expressed concerns about this approach. Fear of privacy disclosure was the main reason. For example, it is still possible for others to see the messages after receiving it. Owing to the special nature of MSM population, the difference in public awareness, and the complexity of the public opinion environment, privacy disclosure in this population may cause physical and psychological harm and negative impact on personal life. Once their privacy is leaked, they may face problems such as discrimination, stigma, breakdown of social relationships, and even experience anxiety, depression, and other psychological disorders [38-40]. After receiving a reminder message, users may be likely to quickly close the page to prevent it from being seen by others. There are also some users who may unbind with the backend system in order not to be discovered by their family and friends. However, this may reduce the reminder system’s effectiveness, resulting in poor performances in the midterm and late stages. Thus, one of the most important aspects of system optimization is the privacy protection of the users.

Moreover, as a new developed reminder system for improving PrEP adherence, it does have some problems that were not considered. For instance, what are the attitudes of users toward this daily reminder? We do not have any surveys on this. Is the frequency of the daily reminders feasible? Would it be more effective to have a weekly reminder instead? It emphasizes the necessity for more personalization. Administrators can communicate with users face-to-face, design reminders to their individual needs, set reminder frequency, and provide feedback and corrections. It was also mentioned in an in-depth qualitative interview that most users showed a strong preference for feedback mechanisms [19]. The ability to respond and receive information may help increase users’ motivation to participate in the program and improve health outcomes. For example, users can interact with the system, and the system will send encouraging words like “Good job” after taking the medicine. In addition, we can try to include health information and education in the WeChat reminder messages, for example, the risk of HIV infection if you do not use PrEP consistently. In this way, we can increase the feedback from the system to the users. The comments and suggestions provided by users provide an important basis for understanding messaging preferences and operating procedures, which further help managers optimize text messages and overall implementation methods.

The reminder system has several advantages. First, although the development and management of the reminder system is challenging, the issue of message costs is fully considered compared to SMS text messaging. Many literatures on SMS text messaging usage do not focus on reducing message costs [41,42], while the reminder system we use does not incur any cost as long as it is bound to the backend system for the first time. Second, according to previous studies, users prefer WeChat as a platform for receiving information and interacting with each other [25]. Compared to receiving text messages, app-based reminders are visually and formally more vivid and interesting, which provide a more convenient platform for our reminder system and are more likely to inspire interest and confidence in users. At the same time, preliminary experience with reminder system practice suggests that given the widespread use of the WeChat app compared to smartphones and other internet resources, this reminder method may be a relatively simple, convenient, and a quick tool to support HIV prevention efforts among high-risk populations [17,43,44]. MSM may benefit from a technology-based intervention that can also be integrated into routine HIV education for high-risk populations. In addition to PrEP, adherence also plays an important role in areas such as HIV antiretroviral therapy, clinical research in traditional Chinese medicine, and chronic diseases [45-47]. Therefore, there is no denying the importance and potential applicability of this reminder system for medicine adherence. The usage of a reminder system not only promotes healthy medicine-taking habits but also decreases the possibility of privacy breaches and costs associated with long-term use. This system is expected to be used on a broader scale after the improvements and upgrades, with a focus on evaluating social and economic benefits as well as targeted dissemination of health education.

### Limitations

There were some limitations in this study. First, adherence was measured by self-report. Owing to recall bias, inaccurate reporting of adherence may occur [48,49]. However, it may also have a social desirability bias, which will cause adherence to be overestimated [50,51]. A previous study has also pointed out that self-reported data may be reliable [52]. Second, the initial design protocol for our study was a prospective cohort study based on a randomized controlled trial in which study participants were randomized to a no-reminder group and a reminder group, and they started using the reminder system once they entered the cohort. However, owing to the impact of COVID-19, the reminder system’s development was delayed, and a portion of the MSM was already recruited. Because of the particularity and privacy of the MSM, finding eligible participants was very difficult. To avoid attrition of the study participants, we had to change the protocol to include the first 3 months of follow-up as the observation period without using a reminder system. When the first 3-month follow-up visit was conducted, the reminder system was initiated, which addressed the problem of the reminder system’s development delay. Nevertheless, this could have a potential impact on our results. Since the study participants took the medicine 3 months in advance, the efficacy of the reminder system was underestimated as they may have developed good adherence during this time. In addition, for the reminder group, we did not ask more questions about the reminder messages. For example, did the participants read the reminder messages every day? Therefore, we were unable to perform a subgroup analysis to assess the effect of different groups. Future studies could focus on more detailed subgroups. Lastly, the specificity of MSM population and the impact of COVID-19 posed significant challenges in recruitment and the follow-up for our study; therefore, longitudinal cohort maintenance in this population was not optimistic.

### Conclusions

Our study constructed a longitudinal cohort of PrEP adherence, explored the factors associated with changes in adherence, and further confirmed the effect of the WeChat-based reminder system on improving PrEP adherence in the MSM population in Western China. The effectiveness of the reminder system in improving adherence is currently significant in the early stage, which is considered to be related to the increased motivation of the users and the development of medicine-taking habits. Although it still can be improved and upgraded, there is no denying the importance and potential applicability of this reminder system for early-stage medicine management, which can help users build medicine-taking habits and increase adherence. Meanwhile, enhancing the privacy protection of reminder system and providing more personalized services and informative feedback are priorities for future studies, which will help improve adherence in the MSM population and promote PrEP implementation.

## Abbreviations

MSM: men who have sex with men
OR: odds ratio
PrEP: pre-exposure prophylaxis
